# Unwrapping the genomic characteristics of urothelial bladder cancer and successes with immune checkpoint blockade therapy

**DOI:** 10.1038/s41389-017-0013-7

**Published:** 2018-01-23

**Authors:** Wen Cheng, Dian Fu, Feng Xu, Zhengyu Zhang

**Affiliations:** Department of Urology, Nanjing Jinling Hospital, Nanjing University School of Medicine, Nanjing, 210002 China

## Abstract

Urothelial bladder cancer (UBC) is one of the most common lethal cancer worldwide and the 5-year survival rate has not improved significantly with current treatment protocols during the last decade. Intravesical immunotherapy with Bacillus Calmette-Guérin is currently the standard care for non-muscle invasive UBC. Recently, a subset of patients with locally advanced or metastatic UBC have responded to checkpoint blockade immunotherapy against the programmed cell death 1 protein (PD-1) or its ligand (PD-L1) or the cytotoxic T-lymphocyte antigen 4 that releases the inhibition of T cells, the remarkable clinical efficacy on UBC has brought total five checkpoint inhibitors approved by the FDA in the last 2 years, and this is revolutionizing treatment of advanced UBC. We discuss the rationale for immunotherapy in bladder cancer, progress with blocking the PD-1/PD-L1 pathway for UBC treatment, and ongoing clinical trials. We highlight the complexity of the interactions between cancer cells and the immune system, the genomic basis for response to checkpoint blockade immunotherapy, and potential biomarkers for predicting immunotherapeutic response.

## Introduction

**Fig. 1:**
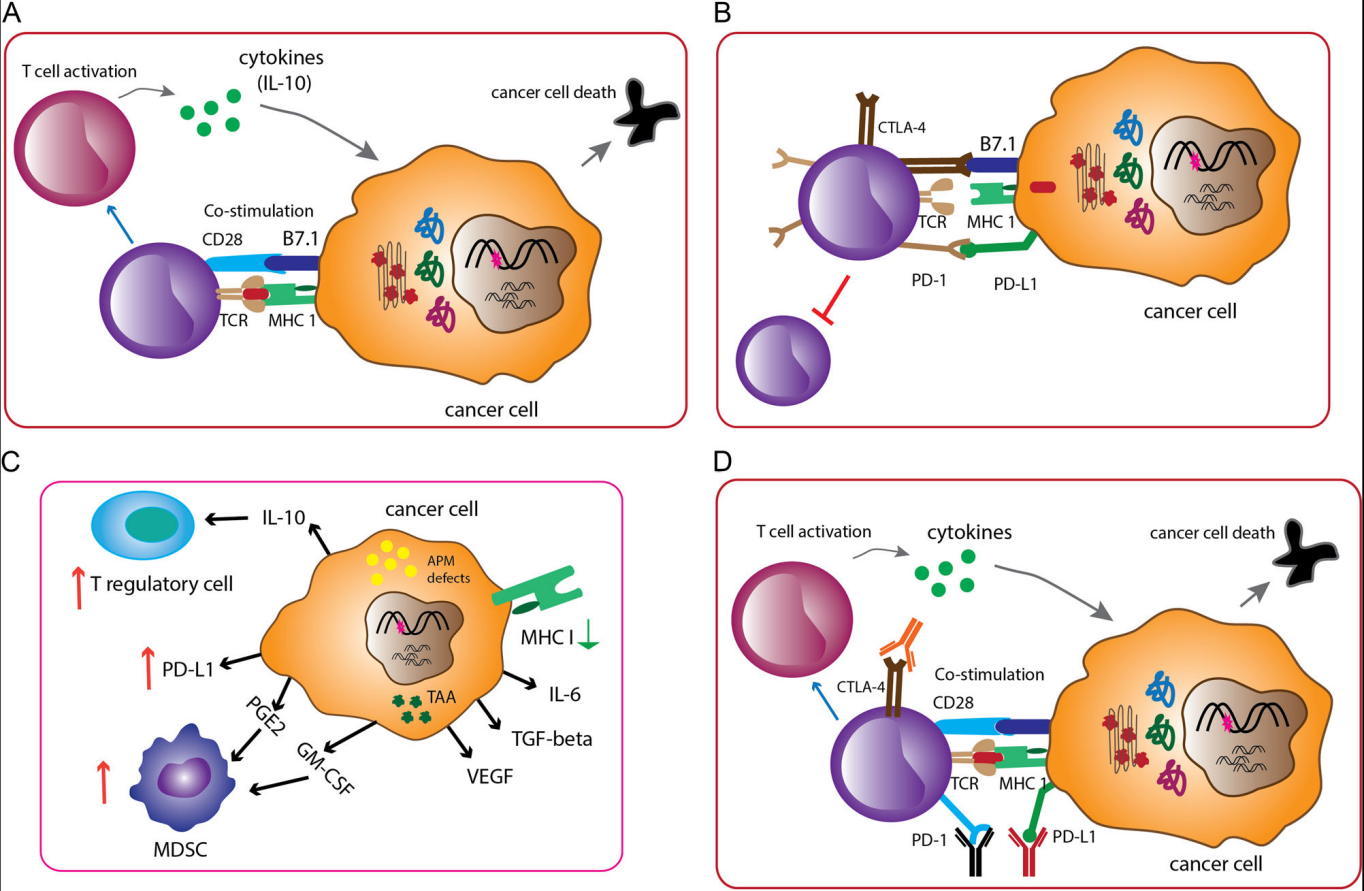
Regulation of T-cell responses and the interaction of cancer cells with host immune responses. **a** Naive T-cell activation takes place after T-cell receptors recognize the major histocompatibility complex (MHC) presenting a specific tumor antigen (signal 1), and the interaction of between CD28 and B7 molecules (CD80 and CD86) (signal 2) expressed on the T-cell surface and on antigen-presenting cells, respectively. **b** T cells express immune checkpoint proteins such as cytotoxic T-lymphocyte-associated protein 4 (CTLA-4) and programmed death/programmed death-ligand-1 (PD-1/PD-L1). CTLA-4 binds B7 molecules with much higher affinity blocking co-stimulation; PD-1 binds the ligand of PD-1 expressed in many cell types, including tumor cells. Both signaling pathways downregulate T-cell responses and protect cells from activated T-cell attack. **c** The complex tumor microenvironment consists of various types of cells, including tumor cells, stromal cells, regulatory T cells, myeloid-derived suppressor cells (MDSC), and inhibitory cytokines, these inhibitory cells abrogate T-cell function and reduce antitumor immune responses. **d** Antibodies against immune checkpoint molecules and increase T-cell responses

The immune system includes both innate and adaptive immunity and it can recognize and destroy malignantly transformed cells. Characteristics of adaptive immunity of the host are highly specific, readily adaptable, and long-term memory response that provides opportunities to treat cancer patients with host own immune system^1^. T-cell activation is followed by interaction between specific T-cell receptor (TCR) and antigen peptides presented by the major histocompatibility complex (MHC), the CD28-B7 co-stimulation increases the binding affinity of the MHC-antigen-TCR complex (Fig. 1a). T cells become activated, then proliferate and differentiate, and release cytokines, such as interferon-γ (IFN-γ), to attack cells expressing specific antigens. Recent research revealed that intrinsic negative feedback signaling presents to control over-reaction of T cells responding to specific antigen stimulation, thus, such T-cell activation also induces inhibitory pathways that eventually attenuate and terminate the T-cell response to keep the immune system in balance. Well-known checkpoint molecules are cytotoxic T-lymphocyte antigen 4 (CTLA-4)^2^, programmed cell death 1 (PD-1)^3^, and PD-1 ligand (PD-L1) (Fig. 1b). The “on” and “off” switcher decides T-cell functions and maintains homeostasis of the immune systems^4^.

**Fig. 2:**
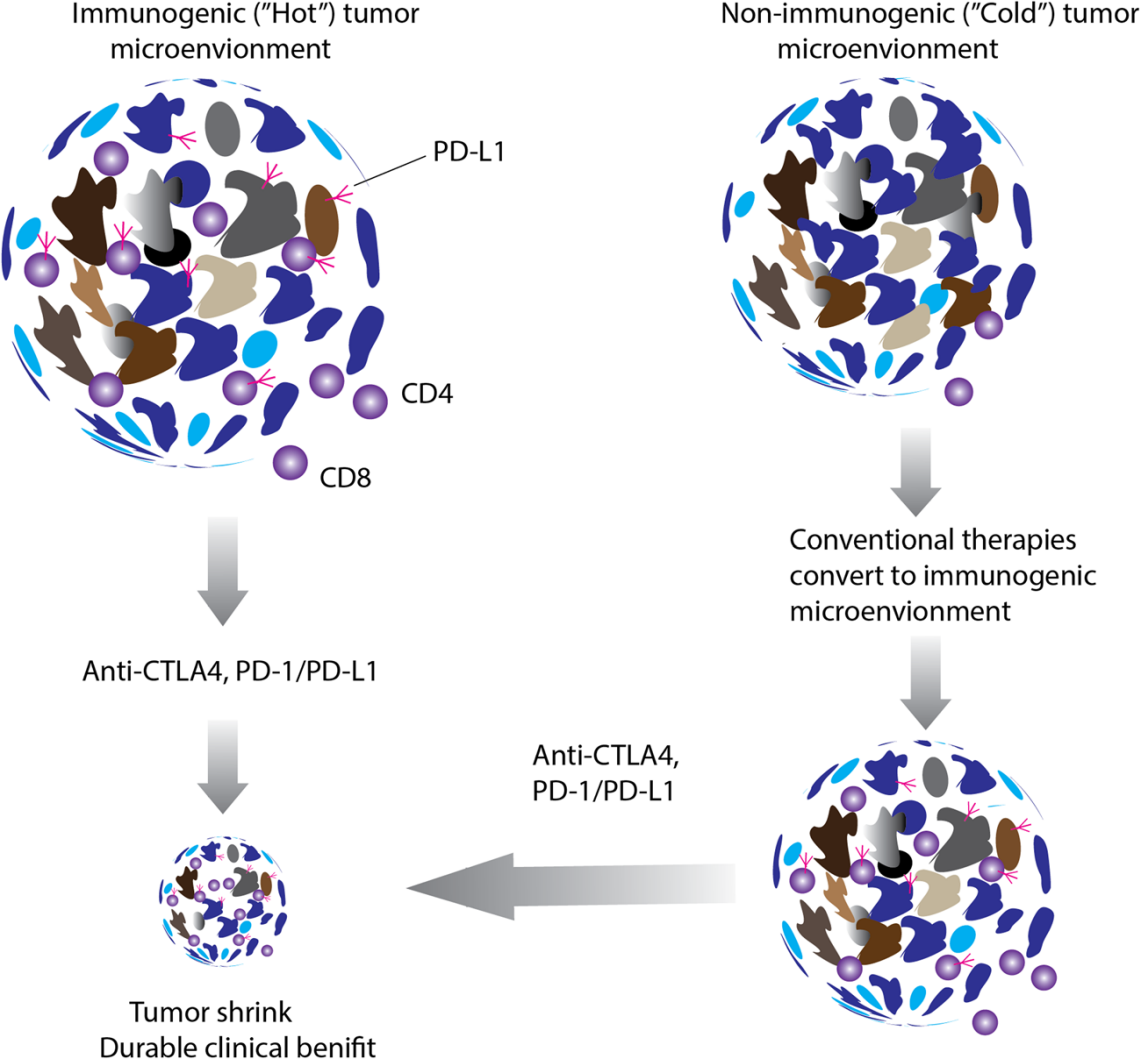
The diagram illustrates the diversity of tumor microenvironment and response to immune blockade inhibitors. Immunogenic tumor microenvironment (left) contains many biomarkers including CD4^+^, CD8^+^ T cells, PD-L1 proteins, and other cells. This “hot” tumor microenvironment with enriched immune cells usually responds to immune checkpoint inhibitors. The “cold”, non-immunogenic tumor microenvironment (right) lacks immune markers and may need combinatory therapeutic modalities to convert the “cold” to a “hot” microenvironment and achieve effective clinical benefit

Immune surveillance is an inherited mechanism by which precursor cancerous cells can be detected as “non-self items” by circulating T cells and B cells, subsequently, the immune cells attack and destroy non-normal cells^5^, but a more complete description of the processes is embodied in the concept of tumor immunoediting. Immunoediting endeavors to provide an annotation of the dynamic interactions between tumor cells and the immune system with three phases: elimination; equilibrium; and escape^1,6^. Success in eliciting activated T cells against tumors is determined by the complexity of the tumor microenvironment (TME), which is an ecosystem of a mixture of different cell types, including, but not limited to, vast majority of tumor cells, scatter of stromal cells, suppressive cytokines, regulatory T cells (Tregs), myeloid-derived suppressor cells, antigens, the expression of MHC molecules, and the expression of PD-L1 by tumors or immune cells (illustrated in Fig. 1c). Hence, the TME can be defined as either immunogenic and “hot” TME or non-immunogenic and “cold” TME according to the amount and content of tumor-infiltrating lymphocytes and expression of PD-L1 protein. Monitoring for “hot” and “cold” TMEs with unique biomarkers could be a good indicator to guide treatment^7^ (Fig. 2).

Cancer immunotherapy includes injection of specific cytokines, tumor-associated antigen vaccines, infusion of adoptive autologous T-cells or genetically engineered T-cells, or immune checkpoint blockades. Recently, specific neoantigens produced from somatic mutations during tumorigenesis have been shown to induce a highly selective T-cell response^8^ and they are emerging as attractive therapeutic targets. Nevertheless, the goal of different treatment modalities is to enhance the activity of the immune system to increase its own natural defense mechanisms against cancer.

Immunotherapy has long history in the course of bladder cancer treatment, and it has played an essential role in the treatment of non-muscle invasive bladder cancer (NMIBC) along with the use of Bacillus Calmette-Guerin (BCG)^9^. The striking improvement in knowledge of immunology has led to the identification of immune checkpoint molecules (such as CTLA-4 and PD-1), whose blockade enhances antitumor immunity in many types of cancers^10,11^ (Fig. 1d). The application of immune checkpoint inhibitors in late stage of bladder cancer has started a new era to manage advanced bladder cancer. The US Food and Drug Administration (FDA) has approved five immunotherapy agents as a second-line or first-line treatment for patients with advanced bladder cancer who either failed to conventional chemo-drug therapy or ineligible to standard protocol since 2016^12,13^. However, the variable response rates in different type of cancers have motivated the search for biomarkers in order to stratify specific patients. Therefore, we will discuss the genomic basis for bladder cancer immunotherapy in this review and update the current status of immunotherapy in bladder cancer. Also, we will highlight genomic biomarkers for predicting response in immune checkpoint blockade therapy.

## Genomic characteristics of urothelial bladder cancer

Urothelial bladder cancer (UBC) is one of the most common cancer of the urinary tract worldwide. It is more common in male than in female (ratio is 3~4:1) and it is characterized by a high rate of relapse, metastasis, and mortality^14^. Median survival time for patients with recurrent or metastatic bladder cancer remains at 14–15 months with cisplatin-based chemotherapy, but there is no widely recognized effective second-line therapy to improve the overall survival time^15,16^. Recently, PD-L1 inhibitors, atezolizumab, durvalumab, and avelumab, and PD-1 inhibitors, pembrolizumab and nivolumab, have been approved by the FDA to treat patients with advanced or metastatic UBC^13,17–19^. The clinical studies for the FDA approval showed overall objective response rate (regardless of PD-L1 expression) is between 13 and 24%, thus the challenging question is why some patients respond to checkpoint inhibitor treatment, others do not. Early studies show that genomic instability of melanoma and non-small cell lung cancer (NSCLC) is associated with the response to checkpoint inhibitor treatment^20^. As with other types of cancer, genomic and epigenomic alterations in urothelial cells are the driver forces in UBC pathogenesis. Therefore, it is speculated that genomic features of UBC could also be responsible for a satisfactory response to PD-L1/PD-1 inhibitors as well as to other novel immunotherapy agents in ongoing clinical trials.

What we have learned from UBC genomic studies? First, urothelial bladder cancer ranks as has having one of the highest mutation burdens among all types of cancer. The mean somatic mutation rate is of 7.7 per megabase listed after melanoma and NSCLC^21^, more than 30% of bladder cancer patients have nonsynonymous mutations above 192 mutations as the threshold in melanoma and NSCLC^22^. On average, there are 204 segmental alterations in genomic copy number, 302 nonsynonymous gene mutations, and 22 genomic rearrangements in each sample, and the UBC-specific known genes *CDKN1A* (P21), *EGCC2* (XPD), *RXRA*, *ELF3* (E74 like ETS transcription factor 3), *KLF5* (transcription factor), *FOXQ1* (forkhead box protein Q1), *RHOB* (Rho-related GTP-binding protein RhoB), *PAIP1* (polyadenlate-binding protein-interacting protein 1), and *BTG* (B-cell translocation gene) are significantly mutated at >3% frequency^21^. Second, several chromatin remodeling genes such as *KDM6A* (lysine demethylase 6A), *CREBBP*, *EP300*, and *ARID1A* (AT-rich interactive domain protein 1A) are highly mutated in bladder cancer^23–25^. The Cancer Genome Atlas (TCGA) study of UBC found that genes regulating chromatin remodeling are more frequently mutated in UBC than in other types of cancer^21^. Third, chromosomal rearrangements (e.g., *FGFR3*–*TACC3* fusion gene) and viral integration (e.g., HPV16) are recurrent structural variants in UBC. These genomic alterations not only change the hallmarks of cancer fundamental cellular pathways such as the p53/RB cell cycle pathway, the RTK/PI3K/mTOR proliferative pathway, and the histone modification chromatin regulatory network, which become potential druggable targets for UBC^25^, but they also produce many non-self, or “foreign” proteins, which could be recognized by activated effector T cells and potentiate cancer cells responding to immune checkpoint inhibitors^26^. Moreover, neoantigens produced from cancer somatic mutations are positively associated with response to anti-PD-1 or anti-CTLA-4 treatment^27^. PD-L1 expression tends to correlate with greater treatment response, but is not a perfect biomarker because bladder cancer with low or no PD-L1 expression has robust response efficacy and better tolerability than traditional chemotherapy^13^. Therefore, genomic characteristics of bladder cancer, just as those of melanoma and NSCLC, may explain the good clinical benefit resulting from immune checkpoint blockade therapy and this has been supported in a recent phase II trial of atezolizumab as a tentative first-line therapy in cisplatin-ineligible UBC patients with locally metastatic UBC^17^.

## Immunotherapy in bladder cancer

Bladder cancer immunotherapy demonstrated beneficial clinical outcome from BCG-treated early stage of UBC and motivated by striking therapeutic efficacy on advanced melanoma and NSCLC with checkpoint blockades. Hereafter, we mainly discuss immune checkpoint inhibitors. Immuno-checkpoint blockades are targeting molecules that express on tumor cells or immune cells, these molecules serve as “brakes” and stop the effective functions of T cells. Treatments with checkpoint inhibitors are developed to release the “brakes” and in turn promote pre-existing anticancer immune responses. The impressive antitumor responses to checkpoint blockades of CTLA-4, PD-1, and PD-L1 have been seen in metastatic melanoma^10^, advanced NSCLC, renal cancer^11^, as well as in advanced bladder cancer^12,13^.

### Immunotherapy for NMIBC

The mainstream of treatment for NMIBC is the complete resection of a tumor followed by induction and maintenance of immunotherapy with intravesical BCG vaccine or intravesical chemotherapy^28^. Intravesical instillation of BCG immunotherapy for intermediate- to high-risk NMIBC could reach as high as 60 – 70% response rates with a long-term therapeutic efficacy^29^. Despite the successful BCG immunotherapy for high-risk patients with NMIBC, ~30% of patients do not respond to the treatment. The evidence of TME seems to influence the therapeutic response to BCG. Tumors from BCG failure present few effector cells and more suppressive immune cells, for an example, CD4^+^ subpopulation and GATA3^+^ T cells (a master regulator of T helper 2-cell differentiation) are scarce in a tumor; expression of FOXP3^+^ (forkhead box P3, also known as scurfin) and CD25^+^ Tregs as well as CD68^+^ and CD163^+^ (markers of M2 macrophage) tumor-associated macrophages are high in TME^30^. This evolving complex ecology of cells results in non-response to BCG treatment. Therefore, combination of BCG with other approaches may change the TME and host immunogenicity. For instance, BCG plus oncolytic adenovirus therapy may be a choice^31^ because oncolytic virus uses a modified, viable-reduced virus that can cause tumor cells to self-destruct and generate a greater immune response against cancer. Administration of immunological checkpoint blockades or other immune modulators to remove inhibitory effects on tumor cells or immune cells is a reasonable strategy. One study indicated that the combination of CTLA-4 molecule blockade with standard BCG therapy could potentiate patients’ immunological activities and ameliorate clinical outcomes of NMIBC^32^. Ongoing clinical trials with checkpoint inhibitors (e.g., pembrolizumab + BCG, trial ID: NCT 02324582) and other novel agents in BCG-refractory patients would provide clinical benefits (Table 1). More details are also found in a recent review paper^33^.

**Table 1:** Developing immunotherapy for advanced bladder cancer

Type	Agents	Clinical trial	Phase
Monoclonal antibodes	Ramucirumab	NCT02426125	III
	B-701, anti-FGFR3 Ab	NCT02401542	II
	MK-6018	NCT02346955	I
	HuMax	NCT02552121	I
Adoptive cell therapy	T cells engineered to recognize the NY-ESO-1, MAGE-A4, PRAME, surviving and SSX markers	NCT02239861	I
Checkpoint inhibitors/immunomodulators	Atezolizumab (PD-L1 Ab, MPDL3280A)	NCT02450331	III
		NCT02662309	II
		NCT02543645	I/II
		NCT01375842	I
		NCT02655822	I
	Durvalumab (MEDI4736):a PD-L1 Ab±	NCT02516241	III
		NCT02527434	II
	Tremelimumab: a CTLA-4 Ab	NCT02643303	I/II
	Nivolumab (Opdivo®): a PD-1 antibody±	NCT02553642	II
		NCT01928394	I/II
	Ipilimumab (Yervoy®): a CTLA-4 antibody	NCT02496208	I
	Pembrolizumab (Keytruda®, MK-3475): a PD-1 antibody	NCT02625961	II
		NCT02500121	II
		NCT02335424	II
		NCT02452424	I/II
		NCT02636036	I
		NCT02437370	I
		NCT02443324	I
Checkpoint inhibitor+chemotherapy	Ipilimumab	NCT01524991	II
	Gemcitabine		
	Cisplatin		
Others	CPI-444 alone	NCT02655822	I/Ib
(adenosine-A2A receptor inhibitor)	CPI-444+atezoliumab		

Adapted from ClinicalTrials.gov

### Immunotherapy for advanced and metastatic bladder cancer

Standard treatment for patients with advanced bladder cancer (e.g., muscle invasive bladder cancer), or metastatic bladder cancer includes cisplatin-based chemotherapy followed by surgical removal of the bladder or radiation therapy and concomitant chemotherapy. Immunotherapy is emerging as an important treatment for patients who do not benefit from first-line chemotherapy, this has been added in revised guideline^34^. Two checkpoint inhibitors (atezolizumab and pembrolizumab) have been used as a tentative first-line treatment in patients with locally advanced or metastatic urothelial carcinoma and patients are cisplatin-ineligible^17,35^. Atezolizumab (MPDL3280A) is a newly developed human anti-PD-L1 antibody that blocks PD-L1 binding with the receptor of PD-1, resulting in PD-1/PD-L1 pathway inactivation. An early clinical trial of atezolizumab observed that patients with advanced UBC respond to anti-PD-L1 antibody often initiated in short time, many cases respond the treatment occurring at 6-week time period, the response rate is ~48%^12^. This expanded phase I study used PD-L1 expression as a robust biomarker to select the subjects for the clinical trial. The results demonstrated that tumors had high response rates if tumor-infiltrating immune cells express PD-L1 proteins. In addition, patients treated with checkpoint inhibitors had less adverse effect compared to cytotoxic chemotherapy^12^. Thus, atezolizumab received accelerated FDA approval in May 2016 for treatment of patients with advanced or metastatic bladder cancer^13^. Subsequent phase II clinical study (single arm and multiple centers) with 315 patients showed a significant objective response rate (14.8%) and durability of clinical response^13^. An additional trial showed atezolizumab could be used as tentative first-line treatment in untreated metastatic urothelial cancer with objective response rate of 23%^17^ and this development is shifting the treatment paradigm for UBC.

Durvalumab is another new PD-L1 inhibitor. Phase I/II trial reported that durvalumab has a manageable safety profile and demonstrated objective response rate of 46.4% in PD-L1-positive patients with metastatic bladder cancer^36^. Based on these promising results, durvalumab is being studied in multiple clinical trials either alone or in combination with CTLA-4 inhibitor, tremelimumab (trial ID: NCT02516241 and NCT02527434). Monotherapy of anti-CTLA-4 (ipilimumab and tremelimumab) or anti-PD-1 (pembrolizumab and nivolumab) are also under clinical trial at different stages (Table 1). Unprecedentedly, in 2017, FDA granted four checkpoint inhibitors, including nivolumab (2 February 2017), durvalumab, avelumab, and pembrolizumab (May 2017) to treat advanced bladder cancer as second-line setting or first-line setting. Selected results, including efficacy, objective response rate, survival time, and adverse events are summarized in Table 2.

**Table 2:** Summary of clinical outcome of different immunocheckpoint inhibitors on urothelial cancer

Study	Phase/indication	ORR or with PD-L1 expression	Survival time	Adverse event	Reference
Atezolumab (PD-L1 inhibitor, NCT01375842)	Phase I/platinum-pretreated	46% (CR 7%, PR 17.6%, IC2/3); 16% (IC0/1)	6 m OS 85% (IC2/3), 71% (IC0/1)	Fatigue, asthenia, and nausea	^69^
Atezolizumab (PD-L1 inhibitor, NCT02108652)	Phase II/platinum-pretreated	15% (CR 5%), 26% (CR 11%, IC2/3); 10% (CR 2%, IC1); 8% (CR 2%, IC0)	Median OS 11.4 m; IC1 6.7 m; IC0 6.4 m	Fatigue, pneumonitis, ASA, AST, rash, dyspnea	^13^
Atezolizumab (PD-L1 inhibitor, NCT02951767)	Phase II/platinum-ineligible, untreated	19%	Median OS 10.6 m	Pruritus, diarrhea, and fatigue. One patient died of sepsis	^17^
Pembrolizumab (PD-1 inhibitor, MK-3475)	Phase Ib/platinum-pretreated	25% (CR 11%, PR 14%)	Median OS 12.7 m	Grades 3 and 4 (15%)	^70^
Pembrolizumab (PD-1 inhibitor, NCT02335424)	Phase II/platinum-ineligible, untreated	24% (CR 6%); 25.4% (CR 6.3%, CPS ≥ 1%); 36.7% (CR 13.3%, CPS ≥ 10%)	Disease control rate ≥ 6 m 83%	Fatigue (14%)	^18^
Pembrolizumab vs. investigators choice (NCT02256436)	Phase III/platinum-pretreated	Pembro: 21.1% (CR 7%); Contl: 11.4% (CR 3.3%)	Median OS: Pembro vs. Contl: 10.7 m vs. 7.4 m (HR 0.73, *p* < 0.05)	Thyroid gland abnormalities, colitis, and pneumonitis	^71^
Durvalumab (PD-L1 inhibitor)	Phase I/II pretreated	31%; 46.4% (CPS ≥ 25%);	3 m disease control rate 57.1% for CPS ≥ 25%; 0% for CPS < 25%	Grade 3 (4.9%)	^36^
				Grade 4 (0%)	
Nivolumab vs nivolumab + ipilimumab	Phase I/platinum-pretreated	24.4%	12 m OS 51.3%	Pneumonitis thrombocytopenia-related death	^19^
Nivolumab (PD-1 inhibitor, NCT02387996)	Phase II/platinum-pretreated	19.9%	Median OS 8.7 m	Diarrhea, fatigue, 1% death	^72^
Avelumab (PD-1 inhibitor, NCT01772004)	Phase I/platinum-pretreated	18.6% (CR 4.5%, PR 9%); 50% (PD-L1+≥5%); 16.6% (PD-L1+>1%)	12 m OS 50.9%	Fatigue, asthenia, nausea, no death	^73^

Tumors are heterogeneous and respond to immunetherapy in dynamic fashion. The expression of inhibitory or stimulatory molecules in the TME is more complexity than we expected. Combination of different checkpoint blockades for poly-immunotherapy is necessary and has demonstrated synergistic or additive beneficial effect in metastatic melanoma^37–39^ and in advanced NSCLC^40^. Therefore, it is reasonable to apply double various checkpoint blockades (checkmates) to treat metastatic bladder cancer with optimized dosages, the sequential order, and good safety. Finally, combining conventional cancer therapies (such as chemotherapy, radiation, surgery, or targeted therapy) and immune checkpoint therapies may improve the response rate and benefit more cancer patients because tumor cell death, caused by conventional therapies, may release neoantigens and initiate T-cell activation, The activated T cells could then travel into tumor tissue, and these immune cells’ functions could be further enhanced by immune checkpoint inhibitors. This phenomenon was observed in patients with chemonaive metastatic UBC in a phase II trial of anti-CTLA-4 (ipilimumab) in combination with chemotherapy (cisplatin and gemcitabine) and measurements showed an increase in circulating CD4^+^ and CD8^+^ T-cell subpopulation in combinatory treatment (NCT01524991)^41^ (Table 1). Therefore, when taken together, approved anti-PD-L1/PD-1 treatment for advanced UBC is providing a great option for subset of patient population. We expect that other ongoing mono- or poly-checkpoint antagonists will also benefit more patients with advanced bladder cancer.

## Predictive biomarkers of response to immunological checkpoint blockades

Remarkable clinical efficacy, durable response, and low toxicity of immune checkpoint blockade treatment have been observed in various malignancies, including UBC^12,13,17,35^. In advanced melanoma, anti-CTLA-4 and anti-PD-1 antibodies have resulted in long-term disease control in a subgroup of patients^10,42^; treatment with antibody targeting PD-1 protein demonstrated objective response rates of 18, 28, and 27% in advanced NSCLC, melanoma, and renal cell cancer, respectively^43^; anti-PD-L1 for advanced bladder cancer could reach 43.3% of response rate in early trial^12^, and >10% of response rate in later large cohort in selected patient population^13,17^. Nevertheless, a large proportion of patients failed to respond to checkpoint inhibitors and, therefore, it is crucial to identify biomarker(s) to stratify or predict responders in order to achieve a better clinical outcome.

The molecular determinants of responsiveness to PD-1/PD-L1 or CTLA-4 inhibitors appear to be heterogeneous and complex due to different mechanisms of action. CTLA-4 functions at early stage, while PD-1/PD-L1 axis functions at late stage of T-cell activation. In 2008, a pilot clinical trial with anti-CTLA-4 (ipilimumab) treatment on 12 early UBC patients before radical cystectomy showed that the expression of inducible co-stimulator (ICOS) had increased significantly in CD4^+^ T-cell subpopulation from both tumor tissues and peripheral blood. The subset of CD4^+^ICOS^hi^ T cells released more effective cytokines (e.g., IFN-γ) and the activated immune cells can recognize the cancer/testis antigen^44^. Moreover, the correlation of increasing ICOS^+^ CD4^+^ T-cell subpopulation following anti-CTLA-4 treatment could monitor the effectiveness of CTLA-4 inhibitor^45^. Either enhancing effector and helper T-cell function or depleting Tregs in TME seems to be necessary for the increase in efficacy of CTLA- 4 blockade treatment^46^. Several studies have reported correlations between clinical outcome with anti-CTLA-4 (ipilimumab) and high peripheral blood lymphocyte count^47^, gene expression signature in microenvironment with high inflammation^48,49^, and induction of T-cell diversification and T-cell repertoire evolution^50^. High somatic mutational load is associated with long-term clinical benefit of CTLA-4 blockage treatment in advanced melanoma, and somatic neoepitope load correlates with responsiveness to CTLA-4 blockade^51^. The patients with metastatic urothelial cancer treated with atezolizumab have better response if they have higher somatic mutational burden^13,17^. The median mutation load was significantly increased in atezolizumab-responders (12.4 mutations/Mb/patient) compared to non-responders (6.4 mutations/Mb/patient). More strikingly, the relationship between somatic mutation load and response was unrelated to TCGA urothelial cancer subtype or PD-L1 expression subgroup^13^. The same cohort was reanalyzed with comprehensive multi-omic data and revealed that higher percentage of TIL and higher peripheral blood TCR clonal expansion are positively associated to durable clinical response to atezolizumab treatment, but whole exome sequencing (not targeted sequencing) of tumor samples did not find the correlation between mutational load, mutation-derived neoantigen load and durable clinical response to atezolizumab treatment, it is possible due to small sample size^52^. Whether mutational burden in bladder cancer could be used as an independent predictive parameter for checkpoint inhibitor therapy remains to be validated with large cohort. Mutational epitopes were positively related to CTL infiltration and amount of memory T cells within a tumor^53^. The higher density of CD8^+^ infiltration surrounding a tumor was associated with better response of advanced urothelial cancer treated with atezolizumub^13,17^ and suggested that the high load of detected immunogenic mutations may likely predict patients who would respond checkpoint blockage treatment^54^. However, combination of PD-L1 expression, mutational load, and bladder cancer subtype appears to be necessary to depict the complexity of likelihood of response to checkpoint inhibitors in metastatic bladder cancers^13^.

The mechanism of action for the PD-1/PD-L1 pathway is different from CTLA-4 signaling; when PD-L1 protein expresses on tumor cells or immune cells in the TME, this results in inhibition of T-cell function. Therefore, most studies use the expression of PD-L1 protein as a biomarker to select patients for treatment with antibodies targeting the PD-1/PD-L1 pathway^12,13,17,43,55^. In general, the higher the expression of PD-L1 the better is the objective response rate and the survival rate (Table 2). In the phase I study of anti-PD-L1 molecule on metastatic bladder cancer, patients with PD-L1 protein-positive tumors were selected for treatment with anti-PD-L1 mono-antibody if more than 5% of PD-L1^+^ tumor-infiltrating immune cells were detected in pretreatment archival tumor samples. The objective response rate was 43.3% after a minimum of 6 weeks of follow-up compared to 11.4% response rate for patients with PD-L1-negative tumors^12^. This is consistent with two other trials of atezolizumab^13,17^ and for different checkpoint inhibitors (e.g., nivolumab, durvalumab, and pembrolizumab in Table 2).

Although PD-L1 protein expression in tumor tissues is correlated with relative higher response rates (Table 2), we still have paradox phenomenon that many tumors that were detected to be PD-L1-positive, indeed, do not respond to treatment, and some patients, whose tumors were PD-L1-negative, had a clinical response to anti-PD-L1 treatment with either tumor reduction or tumor stabilization^56^. It is also worth mentioning that the survival benefit in nivolumab-treated^19^ or durvalumab-treated^36^ or pembrolizumab-treated^18^ advanced urothelial cancer is not concordant with PD-L1 expression levels. These observations suggest that other biomarkers are needed to provide a more accurate and fine-grained prediction. The combination of PD-L1 protein expression and infiltration of T cells was found to have a better overall survival in contrast to tumors with sole one of the features or absent of both features^57^. In urothelial cancer, high PD-L1 expression level is associated with multiple factors such as high expression of C-X-C motif chemokine ligand 9, C-X-C motif chemokine ligand 10, CD8A (indicating activating cytotoxic T cells), and basal subtypes (cluster III and IV), but the best response to atezolizumab is more strongly associated with high CD8+ infiltration in tumor and luminal (cluster II) subtype^13^.

At a genomic level, the overall somatic mutational load has been correlated with clinical response to PD-1 blockade treatment in NSCLC^20^ and in colon cancer harboring mismatch repair deficiency^58^. Consistent with this notion, patients with higher tumor mutational burden have better response to atezolizumab, regardless of subtype of urothelial cancer^13,17^, indicating that tumor mutational burden may have an independent role in predicting response to atezolizumab treatment in urothelial cancer. Furthermore, several studies suggest that neoantigen load^42,51^ or, more precisely, the clonal neoantigen load^59^, is significantly associated with the responsiveness to anti-PD-1. Moreover, important somatic gene mutations in DNA repair^60^, in homologous recombination (BRCA1 mutation), and in replication processes have been identified by their correlation with the responsiveness to anti-PD-1^20^.

Except for their genetic basis, transcriptional signatures are also potential predictors for responding to antagonists of PD-1 molecule. For instance, upregulation of immunosuppressive genes, monocyte and macrophage chemotactic genes (Chemokine (C-C) motif ligand (*CCL*) *2/7/8/13*)), and mesenchymal transition genes (*FAP* (fibroblast activation protein alpha), *AXL* (tyrosine-protein kinase receptor), *ROR2* (receptor tyrosine kinase-like orphan receptor 2), *TAGLN* (transgelin), *WNT5A* (Wnt family member 5 A), *LOXL2* (lysyl oxidase-like 2), *TWIST2* (Twist family BHLH transcription factor 2)) are preferentially in non-responding tumors^42^, and indicate patients with expression of these signatures are most likely to not respond to anti-PD-1 treatment. These findings highlight the complex interplay between cancer cells, the immune system, and other cells in TME, which is ubiquitous in all types of cancer, including urothelial bladder cancer.

## Conclusion and prospects

Modulating inhibitory pathways on immune cells has been a recent major breakthrough in cancer treatment. Immune checkpoint blockade therapy targeting PD-1, PD-L1, and CTLA-4 proteins using specific humanized antibodies has demonstrated good clinical response, long-term disease control, and improved survival time in subsets of patients with advanced melanoma, metastatic NSCLC, metastatic renal cell carcinoma, and advanced bladder cancer. We are expecting more positive clinical data from ongoing clinical trials with combinations of different checkpoint blockades or checkpoint inhibitors with conventional cancer therapies. Since many other molecules are in the T-cell regulatory networks, researchers are actively developing novel antagonists to target other inhibitory pathways, such as lymphocyte-activation gene 3^61^, T-cell immunoglobulin domain and mucin domain 3^62^, V-domain Ig suppressor of T-cell activation^63^, and B- and T-lymphocyte attentuator^64^, as well as agonists to enhance stimulatory pathways, such as ICOS^65^, CD134^66^, and CD137^67^. More recent novel immunotherapy drugs (e.g., ALT-801 (a tumor-targeted IL-2) and ALT-803 (an IL-15 superagonist complex)) have been tested in bladder cancer with promising antitumor activity. More importantly, as we start to understand the genetic and non-genetic basis of immunotherapy responders, we are seeing the potential predictors for such treatment.

It is also worth to mention that compelling data suggest that immunogenic neoantigens and recognition of neoantigens by cytotoxic T cells is a proxy of immunoresponse in clinic. Personalized neoantigen-based vaccination in combination with immune blockade inhibitors is speculated to have better clinical benefits in various solid tumors^60^. More recently, attractive strategy in solid tumors is to redirect neoantigen-specific TCRs into naive T cells for adoptive T-cell infusion and the utilization of chimeric antigen receptor-redirected cytolytic T cells to specifically target the epitope on cancer cells^68^. On the other hand, cancer cells can escape immune surveillance and, under selective pressure, HLA gene mutations, particularly at TCR-binding domains, and other mutations in antigen-presentation-machinery genes have been detected at high level in tumors that are rich in tumor-infiltrating lymphocytes^53^. Therefore, overall, we still face unique challenges to develop completely predictive and prognostic biomarkers for precision bladder cancer immunotherapy.
